# Implementation of artificial intelligence in thoracic imaging—a what, how, and why guide from the European Society of Thoracic Imaging (ESTI)

**DOI:** 10.1007/s00330-023-09409-2

**Published:** 2023-02-02

**Authors:** Fergus Gleeson, Marie-Pierre Revel, Jürgen Biederer, Anna Rita Larici, Katharina Martini, Thomas Frauenfelder, Nicholas Screaton, Helmut Prosch, Annemiek Snoeckx, Nicola Sverzellati, Benoit Ghaye, Anagha P. Parkar

**Affiliations:** 1 Department of Oncology, University of Oxford, Oxford, UK; 2 Department of Radiology, Cochin Hospital, Université Paris Cité, Paris, France; 3 Department of Diagnostic and Interventional Radiology, University Hospital of Heidelberg, Heidelberg, Germany; 4 German Lung Research Center (DZL), Translational Lung Research Center Heidelberg (TLRC), Im Neuenheimer Feld 430, 69120 Heidelberg, Germany; 5 Faculty of Medicine, University of Latvia, Raina Bulvaris 19, Riga, 1586 Latvia; 6 Faculty of Medicine, Christian-Albrechts-Universität zu Kiel, 24098 Kiel, Germany; 7 Department of Radiological and Hematological Sciences, Section of Radiology, Università Cattolica del Sacro Cuore, Rome, Italy; 8 Department of Diagnostic Imaging, Oncological Radiotherapy and Hematology, Fondazione Policlinico Universitario “A. Gemelli” IRCCS, Rome, Italy; 9 Institute of Diagnostic and Interventional Radiology, University Hospital Zurich, Unversity Zurich, Rämistrasse 100, 8091 Zurich, Switzerland; 10 Department of Radiology, Royal Papworth Hospital, Cambridge, UK; 11 Department of Biomedical Imaging and Image-Guided Therapy, Medical University of Vienna, Vienna, Austria; 12 Department of Radiology, Antwerp University Hospital and University of Antwerp, Antwerp, Belgium; 13 Scienze Radiologiche, Department of Medicine and Surgery, University of Parma, Parma, Italy; 14 Department of Radiology, Cliniques Universitaires Saint Luc, Catholic University of Louvain, Brussels, Belgium; 15 Department of Radiology, Haraldsplass Deaconess Hospital, Bergen, Norway; 16 Department of Clinical Medicine, Faculty of Medicine and Dentistry, University of Bergen, Bergen, Norway

**Keywords:** Artificial intelligence, Thorax, Diagnosis, Computer assisted

## Abstract

**Abstract:**

This statement from the European Society of Thoracic imaging (ESTI) explains and summarises the essentials for understanding and implementing Artificial intelligence (AI) in clinical practice in thoracic radiology departments. This document discusses the current AI scientific evidence in thoracic imaging, its potential clinical utility, implementation and costs, training requirements and validation, its’ effect on the training of new radiologists, post-implementation issues, and medico-legal and ethical issues. All these issues have to be addressed and overcome, for AI to become implemented clinically in thoracic radiology.

**Key Points:**

• *Assessing the datasets used for training and validation of the AI system is essential.*

• *A departmental strategy and business plan which includes continuing quality assurance of AI system and a sustainable financial plan is important for successful implementation.*

• *Awareness of the negative effect on training of new radiologists is vital.*

AI is the area of computer science dedicated to creating solutions to perform complex tasks that would normally require human intelligence, by mimicking human brain functioning [1, 2].

Machine learning (ML) is a subcategory of AI in which algorithms perform activities by learning patterns from data, without the need for explicit programming, and which improve with experience [1–3]. ML algorithms are trained to perform tasks based on features defined by humans, rather than statistical instruments organising the data [1–4]. Deep learning (DL) is a subdiscipline of ML that does not require hand-engineered features, but uses multiple hierarchical interconnected layers of algorithms (artificial neural networks), to independently extract and learn the best features, whether known or unknown, to reach a predefined outcome [1, 4]. Further definitions can be studied in the NHS AI dictionary [5].

## Assessment of the evidence

AI is a promising technology in thoracic imaging. Its potential applications are widespread and include improved image noise and radiation dose reduction; AI-based triage and work list prioritization; automated lesion detection segmentation and volumetry; quantification of lesions spatial distribution; and diagnosis support. The scientific evidence for most applications is currently insufficient or lacking, though some applications are commercially available [6]. Abundant evidence for accuracy and precision of AI exists. But with few clinical studies, data on their efficacy and impact on patient care are currently limited, albeit this is emerging [7].

An analysis of 100 CE-marked (Conformité Européenne) AI applications for clinical radiology revealed that for 64/100, there was no scientific proof of product efficacy [6].

During the COVID-19 pandemic countless models of diagnosis support and severity prediction were developed and published. However, several critical appraisals of the literature showed that most AI models suffered from systematic errors [8–10]. A systematic review of 62 published studies on ML to detect and/or prognosticate COVID-19 revealed that none were without methodological flaws or biases, consequently none of the studies were deemed clinically useful [8]. Biases, related to the included participants, predictors, or the outcome variables in the training data were identified. These flaws arose from the limited availability of well-curated multi-centric data for validation. Several guidelines and checklists have been published over the last few years which may assist researchers to perform high-quality studies [11–15]. As AI models enter clinical use, systematic approaches to assess their efficacies become relevant [7]. A list of AI-specific topics to be defined when planning and performing AI studies is given in Table 1.

**Table 1:** Specific issues to remember when planning and writing an AI paper

AI model	Deep learning or machine learning
Aim	Detection, diagnosis or prediction
Function	Stand alone or supportive function
Training sample	
Size	Adequately large > 1000 s
Demographics	Should be included in detail, so that any biases are evident
Training method	How was the gold standard created
	Labelling by radiologists
	Natural language processing
	Extraction from structured reports
	Eye-tracking and report post-processing
Validation method	Retrospective- against a validated database
	Prospectively- against a radiologist

## Potential clinical utility

AI is meant to work alongside radiologists and other clinicians providing relief from tedious and time-consuming tasks on one hand and improving diagnostic accuracy on the other, by integrating complex information faster and more efficiently than is currently possible. AI solutions may contribute to all steps of diagnostic imaging; examination level, reading and reporting, integrating imaging findings with clinical data, and finally at the level of larger patient cohorts [16].

**Fig. 1:**
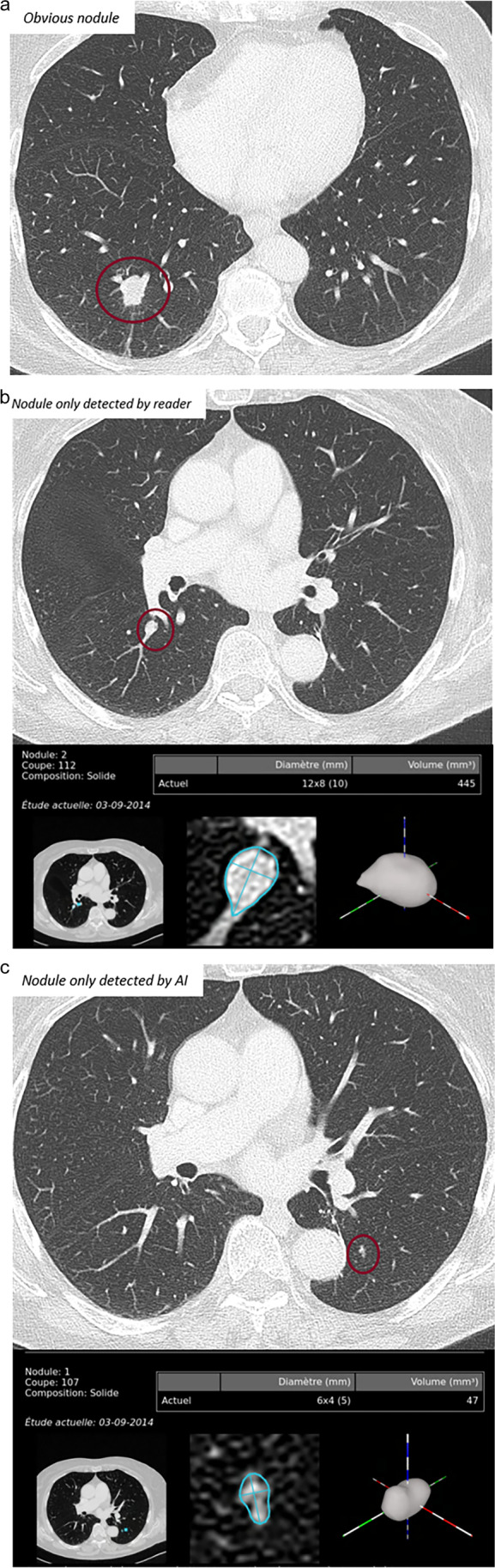
**a**–**c**.Three cases, where the nodule is obvious and detected by both the reader and the AI system (**a**); the nodule is detected by the reader, but not the AI system (**b**); the nodule is detected by the AI system, but not the reader (**c**). (Images courtesy of Prof. Marie-Pierre Revel, Paris)

For reading and reporting AI is meant to augment and/or assist radiologists with automatic detection, feature characterization, and measurements [16]. In thoracic imaging, computer-aided detection (CAD) tools (also known as first-generation AI) have been available for decades now. CAD tools may perform lung nodule detection, interstitial lung disease pattern recognition, and complex analyses of lung emphysema and the tracheobronchial tree. AI has significantly enhanced the performance of said systems [17]. However, acceptance in the hospital/radiology community is surprisingly low. In the arena of lung nodule detection, CAD used as a secondary reader yields significantly superior performance compared to the radiologist’s interpretation alone [18] whilst significantly reducing inter-observer-variability of nodule metrics [19] (Fig. 1). As experienced radiologists have acceptable sensitivity, this step is considered a “time-thief”, as sorting out false-positives and spending time on lesions of questionable clinical importance slows productivity. On the other hand, Martini et al. showed, in a study where they evaluated reading performance in nodule detection with and without the use of CAD software, that the use of CAD led to higher sensitivity and slower reading times in the evaluation of pulmonary nodules, despite a relatively high number of false positive findings [20]. Altogether, automated lung nodule detection using CAD is relevant and essential in lung cancer screening, where maximising nodule detection is critical [21–23].

For most other applications, such as aided interpretation of chest radiographs or quantification of interstitial lung disease, acceptance will depend on their clinical utility and financial footprint. A system that truly speeds up workflow or significantly improves results within the same timeframe or faster than a human reader will be implemented quickly, if they are affordable, but a high-cost AI that adds expense to medical care will be an obstacle [24].

Currently, the radiologist summarizes all findings from different image series or different modalities, visual reviews, and measurements and in some circumstances adds some post-processing steps. The final report reflects the assessment of all components, including written (and sometimes orally received) clinical information, and is approved by the radiologists who take full responsibility for the report. It is likely that a hybrid radiology report containing a combination of radiologist and AI-generated content, such as structured report forms pre-populated by AI algorithms, will become the mainstream [25]. For medicolegal reasons, all contributions from non-human entities must be identifiable. In decision support systems that integrate information from imaging, medical reports, lab results, etc. for the probability of diagnoses, the recommendations change dynamically, as new information is added. Meticulous logs of the AI decision history are needed to connect decisions to a precise date and time. Blockchain-based electronic medical records would allow decisions and contributions to hybrid radiology reports to be traced back, enabling the identification of which version of an AI algorithm contributed and when [13].

The use of AI is not limited to diagnostics but is applied throughout the radiology production chain, starting with planning [26] and image acquisition and processing [27], as well as prioritization of urgent exams for reporting (triage) [28]. AI can also be used for report generation, where image findings are directly incorporated into the clinical report [29].

## Implementation and costs

The successful integration of AI into routine clinical care requires a strategy at the hospital and/or radiology department level. This strategy should define the clinical benefits or organizational goals prior to implementation. The implementation of new AI tools in a hospital involves many varied stakeholders, with established medical routines and professional identities, whilst adhering to strict legal and regulatory standards [30, 31]. Radiologists, hospital managers, and IT members expect there to be significant benefits from the inclusion of AI algorithms into clinical practice [32]. Many vendors promote their products with the promise of improved diagnostic practice, more precise and objective diagnoses, avoidance of mistakes, and the reduction of workload and increased productivity. The latter can only be done by integrating AI into existing IT systems and current workflow practice. AI applications must be implemented into picture archiving and communication systems (PACS) and display understandable outputs with a few clicks. Departments should evaluate the long-term running costs of the system and include this in their business plans.

There are three main types of systems in radiology departments: (1) single workstation, (2) onsite server solution [33], and (3) cloud-based server solution [34]. Single workstations with AI systems are mostly used for research. Many radiological sites use server-based solutions that communicate with PACS providing results either as secondary capture in the PACS or in a web-based application. Cloud-based server solutions function well but include some barriers. Data are uploaded onto cloud platforms after pseudonymisation. The results are returned and connected to the patient’s data with a data-protected internal pseudonymisation key, with issues related to the location of cloud servers and national data protection [35]. A further hurdle is the lack of compensation for the use of AI in radiology, potentially leading to unstructured implementation with high costs, limited efficiency, and poor acceptance among radiologists [36] Table 2.

**Table 2:** Checklist for implementing an AI system in the department

Critical assessment of the training and validation datasets
Examine the formal CE approval
Examine compatibility with existing IT
Assess the purchasing and running costs
Ensure GDPR adherance
Plan training of the users of the AI system, especially for safe use in clinical work
After implementation, confirm that the AI product is working as planned
Confirm that the users can access it as planned
Implement a system for user feedback and regular checks
Plan for the negative effect of AI on the training of new radiologists
Consider the ethics regarding AI, transparency and avoidance of medicalisation

### Integration workflow – reader – reading time

Currently, most commercial algorithms are validated to be used as concurrent or second readers. If used as a concurrent reader, the radiologist has simultaneous access to the results of the AI system, while interpreting the images. As the second reader, the AI system is enabled after the radiologist has read the scan. AI systems as the first reader, where the radiologist only checks the AI results, are not yet licensed for clinical work. Regardless of how much AI performance advances in the future, AI will never replace physicians (see section Medicolegal and ethical concerns).

Data from mammography screening shows that the use of an AI algorithm decreases the reading time for normal cases but slightly increases the reading time for abnormal cases [37]. Data comparing CAD for nodule detection in chest CT as a second reader versus CAD as a concurrent reader is limited, with varying results [38–41]. Several studies have shown that reading time with concurrent CAD is shorter than with CAD as a second reader with minimally poorer performance often with only one task being compared [38, 42, 43]. Muller et al investigated the impact of an AI tool on radiologists reading time for non-contrast chest CT examinations and found that the AI tool did not increase reading time, but in 5/40 cases led to additional actionable findings [44].

### Training and validation

#### Training and supervision

The correct strategy to obtain accurate DL models is to use large, curated and annotated datasets, preferentially derived from multiple institutions in different geographic areas, to ensure the generalizability of the model for clinical use [13].

Such “high-dimensional” data (also called “big data”) are required to avoid “overfitting”, a phenomenon where the AI model learned well not only the valid data, but also the noise, yielding accurate predictions on the training set but failing to perform adequately on new data and different samples. Overfitting is quite common with DL algorithms [4, 45, 46]. “Underfitting” may occur when the DL algorithms demonstrate poor performance on both training and validation sets, due to inherited biases in the training dataset (i.e. multiple insufficiently represented subpopulations, unsatisfactory number of parameters, inadequate nature of the model itself) [4]. Therefore, high reproducibility and robust segmentation of data are crucial. This data has to cover almost the same ratio of characteristics (age, gender, various categories, etc.) for the training and validation cohorts.

Training samples can be increased by data augmentation, consisting of altering the existing images within the training set according to different methods (i.e. random rotation, brightness variation, noise injection, blurring among others), or creating new images (synthetic data augmentation). Even though data augmentation methods can reduce overfitting and increases model performance, they may not be able to capture variants that are found in larger datasets [47].

Federated learning, where the algorithm is trained in different institutions on local datasets, then an aggregated model is sent back and retrained, allows access to a larger and differentiated database, which is particularly useful for uncommon and rare diseases, whilst at the same time reducing patient data confidentiality concerns [47–49]. An additional method to increase database size for AI learning and validation is by direct sharing of radiological examinations and electronic medical records (EMR) by patients themselves and should be encouraged (patient-mediated data sharing) [50].

Apart from a large amount of data, well-curated labelled and annotated datasets are required to train AI algorithms. Labelling is defined as the process of providing a category to an entire image/study (critical in classification tasks), whilst annotation refers to providing information about a specific portion of an image (required for detection, segmentation, and diagnostic purposes) [50]. In supervised methods, labelled or annotated data are needed to reach the outcome, but annotation is a time-consuming task and requires a high level of expertise in radiology. Consequently, large well-annotated databases are not readily available [47]. In semi-supervised methods, a combination of both labelled and unlabelled outputs is sufficient, as the algorithm progressively learns to harness the unlabelled data, reducing the requirement for a vast amount of labels/annotations [4, 47].

Potential strategies to obviate retrospective manual labelling by radiologists depend upon direct extraction of information from EMR (so-called electronic phenotyping) or radiology reports. Structured reporting methods allow the facilitation of this task [50].

A major drawback of any dataset training that extracts diagnoses and findings using natural-language processing, tracking radiologists’ eye movements, and labelling by radiologists may include radiologists’ own mistakes and missed findings. Some authors suggest that only training based on the “gold standard” or “truth” which include diagnosis made by several radiologists or expert panels with adjudicated standards should be used to generate an AI model [35, 36, 45, 46].

#### Validation

Validation data refers to a new set of data used to control or test the already trained AI system [5].

There are two ways the AI systems are usually validated: either retrospectively where the AI must perform as well as the gold standard (known database or radiologists) [51–54] or prospectively where the reader utilises AI as a second reader and the rate of change in reports is the measurement of success [55–60]. Although training may initially be done on large datasets (> 1000 s of unique images), validation is often done on just hundreds of images [55, 60, 61].

### Effect on training of new radiologists

Currently, radiology training includes/requires the trainee to read and evaluate hundreds and sometimes thousands of CT and MRI scans and thousands of radiographs in order to gain experience to evolve into a fully trained radiologist. Supervision is given directly to the trainee with how to approach the imaging, what to look for, and the abnormalities if present explained, and their significance in the context of that patient’s care, all included as an essential part of the learning process. In particular, learning what is normal or an incidental normal variant or variation is critical and takes hundreds or thousands of images to learn. When AI is implemented into a department, one should be aware of how this will affect training. If it is used as a second reader, it will aid in diagnosis immediately, but the trainee may not understand why. AI might be used as a second reader, freeing up specialists for other tasks, but the trainees will lose out on important interactions with their supervisors, potentially resulting in situations where younger on-call radiologists use AI as a first reader (“off-label” use) and base their reports on the AI results. This will invariably affect what older radiologists consider "common or basic knowledge". If the basic radiology training in the future is too weak, then they may have difficulty challenging incorrect predictions made by AI [62]. This effect on radiology may take many years to manifest and by then may be irreversible. Therefore, it has to be emphasized, that AI always will only act as a system that supports radiologists, having the final decision on the report. For future radiologists, it will be crucial to be educated in traditional radiology and the value of AI.

## Post-implementation review

The promise of efficiency gains, cost reduction, and quality improvement are typically foremost in terms of the expected benefits from AI, but its performance reported in research studies may diverge from its performance in routine clinical settings. Measures of quality improvement in imaging are complex without a universally agreed methodology. An ongoing challenge requiring further research is how best to measure the effectiveness of AI in ‘the real world’, whilst also demonstrating sustainable cost-effectiveness. Until there is proof of patient benefits, improved workflow, and reduced costs, a local formal approved institutional innovation strategy for AI is essential.

The predefined metrics which need to be included in the approval for each algorithm should be defined by radiologists working with clinicians who have knowledge of the patient pathway and the significance of each step should be included, in order to safeguard patient care. The intended benefits and potential for unintended consequences should be formally assessed. The AI system provider should also have a routine for picking up the deterioration of the system after being applied in clinical work, especially if the system is dynamic and not fixed [63]. A routine for introducing new diagnostic criteria should also be in place. Without evidence of the proven added value of AI in clinical practice, justification of the required funding for further implementation may be problematic, and the higher costs difficult to defend.

## Medicolegal and ethical concerns

### Medicolegal aspect

The primary objective of legal regulation of the use of AI and ML technology in healthcare is to limit the emergence of risks to public health or safety and to respect the confidentiality of patients’ personal data [64].

The substantial increase in approved AI/ML-based devices in the last decade [65] highlights the urgent need to ensure rigorous regulation of these techniques. To date, the legal regulation of AI/MLs’ performance in healthcare is still in its infancy [64]. Currently, no Europe-wide regulatory pathway for AI/ML-based medical devices exists and is solved by each country individually, adjusted to national legal systems [64, 65].

In Europe and the USA, any algorithm or software which is intended to diagnose, treat, or prevent health problems is defined as a medical device under the Food, Drug, and Cosmetic Act (in the USA) and the Council Directive 93/42/EEC (in EU countries) and needs to be approved by the respective entity [65]. Therefore, hospitals or research institutions utilizing “in-house” developed tools in the clinical routine without approval or CE mark, have to do so with caution [66].

If the software is used as a second reader, the liability is probably on the report-signing radiologist. In the case of stand-alone AI/ML software, this liability might shift to the manufacturer. A grey area is when AI/ML software is used for worklist prioritization. If a potential life-threatening finding/condition is “missed” by AI, the patient might be diagnosed later than with the usual “first in first out” prioritisation, with a serious adverse impact on the patients’ clinical outcome. The other way around, if AI points out a lesion but the radiologist determines that it is not a lesion, there is a possibility of legal liability issues if the radiologists’ judgment deviates from the actual results. Although the current AI may have reached sufficient performance in terms of detection, in qualitative diagnosis, it is desirable to develop an explainable AI that can display the rationale for the diagnostic process.

Two approaches to the legal regulation of the application of AI/ML technologies are discussed in the literature:

- The formal (legal) approach, where the responsibility for the actions of an AI/ML algorithm is assigned to the person who launched it;
- The technological approach, where insurance of liability of an AI/ML technology covers the damage caused by the robot [64].

In 2021, the European Commission published a draft of the world’s first law to regulate the development and use of systems based on AI/ML [64].

Until then, we recommend more transparency on how devices are regulated and approved to improve public trust, efficacy, patient safety, and quality of AI/ML-based systems in radiology [65, 66]. It will also be key to determine where liability lies when an AI company ceases to trade or is purchased by another company, and to ensure that no data may be passed to a third party without individual institutional approval.

### Ethical aspect

Ethics regarding AI is based on the general ethical concepts in radiology that are applicable even today, as stated in the ESR code of ethics [67]. Radiologists must aim to not harm patients, whilst delivering the highest possible care.

The ESR and North American statement on ethics of artificial intelligence in radiology includes patient rights on the use of their data in data sets, biases in labelling, explainability, data safety (protection against malicious interference), quality assurance, automation bias, and accountability of AI systems developers at the same level as physicians [68]. It is desirable that an explainable AI that can display the rationale for the diagnostic process is developed.

Chest radiologists can improve the ethical use of AI software, by demanding transparency of the systems, and examining biases as explained earlier in this paper.

When planning implementation, radiology departments must educate their staff about the technology, the benefits, and potential shortcomings, in order to be able to explain this to patients and clinicians when asked [69].

After implementing an AI system into clinical workflow, the application must be used correctly and as licensed. Unethical use of the system, such as performing a task it is not devised for (no off-label use) or using it as a first reader when it is designed to be a second reader, is unacceptable and may endanger the patient.

There is also an emerging issue that radiologists and clinicians need to face. How to deal with subclinical findings which are detected and or diagnosed by AI? If AI systems find pathology normally invisible to the naked eye, triggering further investigation which might cause additional radiation exposure and medicalisation, in direct contradiction with the “choose wisely” campaign aimed at reducing additional exams [70]. An example is the recent paper showing how AI improved the detection of pneumothorax after biopsies [71]. However, the clinical significance of missed pneumothoraxes was not discussed, as pneumothoraxes < 2 cm in clinically stable patients require no intervention, and the value of higher detection remains questionable [72].

## Summary

**Fig. 2:**
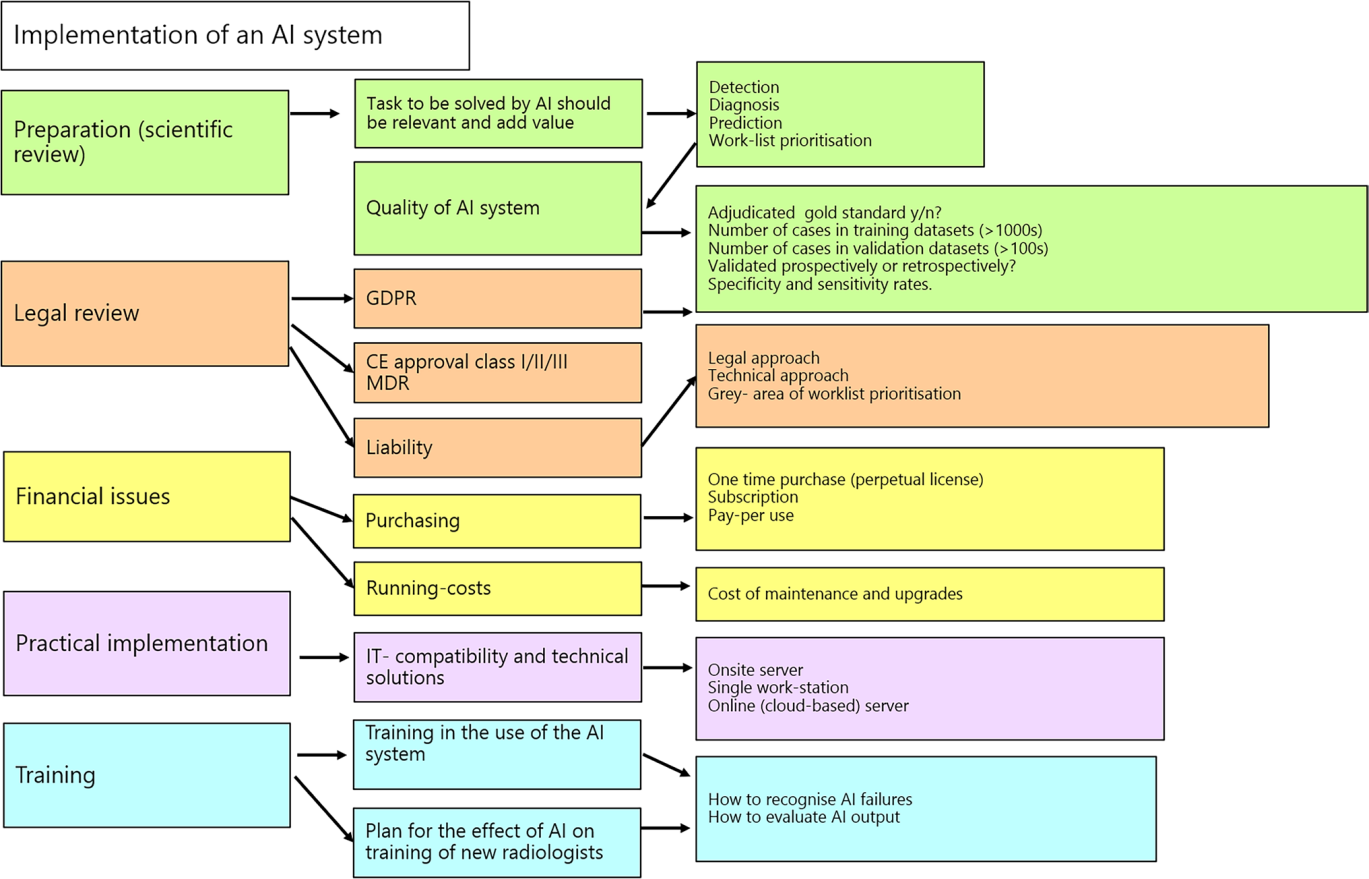
A flow chart for the items to remember when implementing AI in a radiology department

We present a guide for implementing AI in (chest) imaging (Fig. 2).

In summary, the successful implementation of AI in chest radiology requires the following:

- Establishment of a defined department-specific AI strategy, with clear definitions of the role, type, and aim of AI applications in the clinical workflow, continued software quality assurance, and a financially sustainable business plan
- CE/FDA approval
- Critical review of training and validation datasets to ensure they are free from biases and are sufficiently accurate to safeguard patients
- Integration of the AI into existing clinical workflow
- Clarification of ICT requirements and long-term costs with relevant stakeholders
- Knowledge of how patient data is handled in the AI to guarantee data protection in accordance with GDPR
- Proper training of radiologists about its use and limitations
- Awareness of medico-legal issues and liability issues
- Awareness of the potential negative effects of AI on the training of future radiologists in the short and long term

## Conclusions

Broad clinical implementation of AI is on the horizon. Once the systems are good enough for clinical practice, the remaining challenges concerning continued quality assurance, finance, and training of future radiologists will have to be resolved for AI.

## Declarations

### Guarantor

The scientific guarantor of this publication is Anagha P. Parkar.

### Statistics and biometry

No complex statistical methods were necessary for this paper.

### Methodology

- review of current literature
- retrospective
